# Association between prehospital shock index variation and 28-day mortality among patients with septic shock

**DOI:** 10.1186/s12873-022-00645-1

**Published:** 2022-05-19

**Authors:** Romain Jouffroy, Basile Gilbert, Léa Thomas, Emmanuel Bloch-Laine, Patrick Ecollan, Josiane Boularan, Vincent Bounes, Benoit Vivien, Papa-Ngalgou Gueye

**Affiliations:** 1 Intensive Care Unit, Ambroise Paré Hospital, Assistance Publique - Hôpitaux de Paris, Paris, France; 2 IRMES - Institute for Research in Medicine and Epidemiology of Sport, INSEP, Paris, France; 3 INSERM U-1018, Centre de Recherche en Epidémiologie Et Santé Des Populations - U1018 INSERM, Paris Saclay University, Paris, France; 4 Université de Paris, 7329 Paris, EA France; 5 Intensive Care Unit, Anaesthesiology, SAMU, Necker Enfants Malades Hospital, Assistance Publique - Hôpitaux de Paris, Paris, France; 6 Department of Emergency Medicine, University Hospital of Toulouse, SAMU 31, Toulouse, France; 7 Hôpital d‘Instruction Des Armées Bégin, Paris, France; 8 Emergency Department, Cochin Hospital, Paris, France & Emergency Department, SMUR, Hôtel Dieu Hospital, Paris, France; 9 Intensive Care Unit, SMUR, Pitie Salpêtriere Hospital, 47 Boulevard de l‘Hôpital, 75013 Paris, France; 10 Centre Hospitalier Intercommunal Castres-Mazamet, Paris, France; 11 SAMU 972 CHU de Martinique Pierre Zobda Quitman Hospital, Fort-de-France Martinique, France

**Keywords:** Septic shock, Prehospital setting, Mortality, Shock index, Variation

## Abstract

**Purpose:**

Septic shock (SS) hyperdynamic phase is characterized by tachycardia and low-blood pressure reflecting the relative hypovolemia. Shock index (SI), the ratio between heart rate and systolic blood pressure, is a simple objective tool, usable for SS prognosis assessment.

This study aims to evaluate the relationship between prehospital SI variation and 28-day mortality of SS patients initially cared for in prehospital setting by a mobile intensive care unit (mICU).

**Methods:**

From April 6^th^, 2016 to December 31^st^, 2020, 406 patients with SS requiring prehospital mICU were retrospectively analyzed. Initial SI, i.e. first measurement after mICU arrival to the scene, and final SI, i.e. last measurement of the prehospital stage, were used to calculate delta SI (initial SI—final SI) and to define positive and negative delta SI. A survival analysis after propensity score matching compared the 28-day mortality of SS patients with positive/negative delta SI.

**Results:**

The main suspected origins of infection were pulmonary (42%), digestive (25%) and urinary (17%). The 28-day overall mortality reached 29%.

Cox regression analysis revealed a significant association between 28-day mortality and delta SI. A negative delta SI was associated with an increase in mortality (adjusted hazard ratio (HRa) of 1.88 [1.07–3.31] (*p* = 0.03)), whereas a positive delta SI was associated with a mortality decrease (HRa = 0.53 [0.30–0.94] (*p* < 10^–3^)).

**Conclusion:**

Prehospital hemodynamic delta SI among SS patients cared for by a mICU is associated with 28-day mortality. A negative prehospital delta SI could help physicians to identify SS with higher risk of 28-day mortality.

## Introduction

Every year, sepsis affects more than 30 million people worldwide [1–3] leading to around 11 million deaths [3]. Despite progress in the prevention, diagnosis and care, sepsis related mortality rate still ranges from 10 to 20% and from 50 to 60% for septic shock [4–6].

The World Health Assembly, the World Health Organization and the “SEPSIS-3” conference emphasize prevention, early recognition, severity assessment and treatment of septic patients to decrease mortality [7, 8]. Early recognition of septic shock (SS) is the first step prior diagnosis, severity assessment and treatment implementation [7], in both prehospital and in-hospital setting [3, 6]. In the prehospital setting, severity assessment influences the orientation to the appropriate ward, i.e. emergency department (ED) for less severe patients or intensive care unit (ICU) for the most severe patients [9]. In the prehospital setting, the early recognition and prognostication of sepsis remains a daily challenge, most of the time based on clinical examination [10]. Among tools that can be used to recognize and to assess the severity, we previously reported that skin mottling score and capillary refill time, and initial prehospital shock index (SI), ratio between heart rate and systolic blood pressure [11], normal range from 0.5 to 0.7 in healthy adult [11], are associated with increased mortality of patients with SS initially cared for in the prehospital setting [12, 13].

By similarity with the relative blood lactate clearance, which is a prognostic tool usable in the pre and in-hospital setting for sepsis severity assessment [14, 15], for treatment effect assessment [7, 15–18] and to guide sepsis resuscitation [7, 19, 20], we investigated in this study the association between prehospital SI variation and 28-day mortality of patients presenting with septic shock initially cared for in prehospital setting by a mobile intensive care unit (mICU). The aim of the study was to show that, in the same way as lactate clearance, is an indirect tool for treatment effect assessment [7, 21], the change in SI is a clinical tool allowing of treatment effect assessment.

## Methods

### Background

As previously reported [22], the French prehospital emergency medical service (EMS) is based on the SAMU (Urgent Medical Aid Service), a public health control organization, which provides medical response to prehospital emergency situations. The SAMU is made up of emergency physicians and assistants answer calls who respond to the patients' complaints [23]. In the case of life-threatening emergencies, a mICU, the SMUR (Mobile Emergency and Resuscitation Service) staffed with an emergency physician and equipped with medical devices and drugs allowing initial management of major organ deficiency, is dispatched to the scene [24] in order to provide out-of-hospital treatment and transport to definitive in-hospital care, either the ED or the ICU.

### Patients

From April 06^th^, 2016 to December 31^st^, 2020, septic shock patients to the 2012 sepsis-2 conference [25] cared for by a mICU teams of 7 hospital centres (Necker-Enfants Malades Hospital, Lariboisière Hospital, La Pitié-Salpêtrière Hospital, Hôtel Dieu Hospital, APHP, Paris – France; The Paris Fire Brigade Paris, – France; the Toulouse University Health Centre, Toulouse – France and the Castres Hospital, Castres – France), were retrospectively analyzed. Septic shock diagnosis was established after hospital admission and patient identification based on medical hospital reports. In the prehospital setting, septic shock diagnosis was presumed on clinical history, clinical signs and lactate measurement of available according to the sepsis-2 conference [25].

Patients younger than 18 years, and/or pregnant, and/or at the terminal stage of any comorbidity and/or with guardianship or curatorship were not included in this study [26]. One-hundred and fourteen among the 406 patients included in this study were previously retrospectively analyzed [13].

Patients’ demographic characteristics (age, weight, height, and gender), supposed prehospital origin of sepsis, initial prehospital (i.e., the first mICU contact), and final prehospital (i.e., at the end of prehospital stage) vital sign values (systolic (SBP), diastolic (DBP) and mean arterial pressure (MAP)) were measured with a non-invasive automated devices in all centres. Heart rate (HR), pulse oximetry (SpO2), respiratory rate (RR), temperature and Glasgow coma scale (GCS)), plasma blood glucose level, duration of prehospital care, and prehospital treatments delivered (antibiotic therapy type and dose, fluid volume expansion type and dose, as well as catecholamine type and dose), were collected from mICU prehospital medical reports. Previous underlying comorbidities (chronic cardiac failure, chronic renal failure, chronic obstructive pulmonary disease, diabetes mellitus, and history of cancer) were also collected [27].

Initial SI corresponds to the SI measured by mICU after their arrival to the scene, whereas final SI corresponds to the SI measured just prior hospital arrival. Delta SI represents the difference between initial SI and final SI. Delta SI was encoded as a categorial variable (0 for negative delta SI or 1 for positive delta SI).

Initial blood lactate is the blood lactate value measured after mICU arrival to the scene and final blood lactate the value measured just prior hospital arrival using a point of care medical device (StatStrip® Lactates, Nova Biomedical, Waltham, MA, USA) with correct comparability and transferability according to the central laboratory analyzers [28]. Delta lactate was estimated by the following equation: ((initial blood lactate—final blood lactate) / prehospital duration) (mmol.l^−1^.minutes^−1^). Delta lactate was encoded as a categorial variable (0 for negative delta lactate or 1 for positive delta lactate).

The in-hospital length of stay (LOS) and the 28-day mortality were retrieved from medical reports in case of death in hospital or by phone call when the patient was discharged from the hospital. The Sequential Organ Failure Assessment (SOFA) score [29] and the Simplified Acute Physiology Score (SAPS 2) [30] were calculated 24 h after ICU admission.

In order to minimize the bias in data abstraction [31], data collection was performed by a single investigator (RJ) using a standardized abstraction template.

### Ethical considerations

The study was approved by the French Society of Anesthesia and Intensive Care ethics committee on December 12^th^, 2017 (Ref number: IRB 00,010,254–2017-026). The French Society of Anesthesia and Intensive Care ethics committee waived the need of informed consent.

### Statistical Analysis

Results are expressed as mean with standard deviation for quantitative parameters with a normal distribution, as median with interquartile range [Q1-Q3] for parameters with a non-Gaussian distribution, and as absolute value and percentage for qualitative parameters.

The primary outcome was the 28-day mortality rate.

### Statistical analysis was a priori decided, and the statistical analysis plan developed prior to analysis.

Univariate and multivariate analyses were performed to evaluate the relationship between each covariate and the 28-day mortality rate.

To reduce the effect of confounders on 28-day mortality and on delta SI, a propensity score matching was used to balance the differences in baseline characteristics between patients with positive SI and those with negative SI [32]. The propensity score was estimated using logistic regression based on potential confounders on 28-day mortality and on SI variation: age, prehospital duration, prehospital catecholamine infusion [26], prehospital fluid expansion [26], hypertension [26], chronic cardiac failure, chronic renal failure, chronic obstructive pulmonary disease, history of cancer, diabetes mellitus, immunodepression, prehospital antiobiotic administration [9] and hospital length of stay. "Hospital centre" variable was also added in the propensity score in order to take into account the heterogeneity between centres’ practices and the number of patients included by each centre. The nearest neighbour matching method was used to match patients based on the logit of the propensity score [32]. The balance of covariates after matching was assessed by absolute mean differences with a considered acceptable threshold of 15% [33].

Imbalance matching was assessed with standardized mean deviation, based on the following formulae to assess the standardized mean deviation (SMD):

$$SMD=100* \frac{|\mathrm{x }(\mathrm{cases})-\mathrm{x }(\mathrm{controls})|}{\sqrt{{\left(s cases\right)}^{2}+s {\left(controls\right)}^{2}}}$$

where x denotes the mean or proportion for binary variables and classes of categorical variables et s the variance.

Thereafter, in the matched sample, baseline characteristics were compared between cases (patients with negative SI) and controls (patients with positive SI) by paired tests.

Finally, in the propensity score–matched cohort, a survival analysis using Cox proportional hazards regression was used to compare the 28-day mortality of patients according to (i) the positive or negative delta SI, and (ii) the positive or negative delta lactate. Proportional hazards assumption was verified for each Cox model variable by Kaplan Meier curves and the log-rank test.

Results are expressed by adjusted Hazard ratio (HRa) with 95 percent confidence intervals [95 CI].

All tests were 2-sided with a statistically significant *p-value* of < 0 0.05. All analyses were performed using R 3.4.2 (http://www.R-project.org; the R Foundation for Statistical Computing, Vienna, Austria).

## Results

### Population characteristics

Among, the 406 patients analyzed with septic shock requiring action by the mICU, 268 patients (68%) were male, and the mean age was 69 ± 15 years old (*Table **1*). One-hundred and thirty-five patients (33%) were admitted to the ED and 271 patients (67%) admitted to the ICU after mICU intervention.

**Table 1:** Population characteristics. Results were expressed as mean and standard deviation for quantitative parameters (normal distribution), as median and interquartile range for quantitative parameters (non-gaussian distribution) and, as absolute value and percentage for qualitative parameters. P-value corresponds to the comparison between deceased and living patients

	***Overall population (n* = *406)***	***Living (n* = *290)***	***Deceased (n* = *116)***	*p value*
***Demographics***				
Age (years)	69 ± 15	67 ± 16	72 ± 14	**0.005**
Hypertension	166 (41%)	117 (40%)	49 (42%)	0.726
Chronic cardiac failure	69 (17%)	44 (15%)	25 (22%)	0.124
Diabetes Mellitus	106 (26%)	83 (29%)	23 (20%)	0.07
Cancer history	144 (35%)	95 (33%)	49 (42%)	0.072
COPD	55 (14%)	34 (12%)	21 (18%)	0.092
Chronic Renal Failure	52 (13%)	32 (11%)	20 (17%)	0.093
***Prehospital initial values***				
Initial SBP (mmHg)	102 ± 43	103 ± 47	97 ± 27	0.193
Initial DBP (mmHg)	59 ± 20	60 ± 21	58 ± 19	0.240
Initial MBP (mmHg)	72 ± 22	73 ± 23	70 ± 20	0.211
Initial HR (beats.min^−1^)	112 ± 28	112 ± 28	112 ± 30	0.974
Initial SI	1.2 ± 0.5	1.2 ± 0.5	1.2 ± 0.5	0.354
Initial RR (movements.min^−1^)	30 [22-38]	28 [22-36]	32 [25-39]	0.088
Initial pulse oximetry (%)	93 [87 – 96]	94 [88 – 97]	91 [83 – 95]	0.012
Initial body core temperature (°C)	38.5 [37.0 – 39.3]	38.6 [37.1 – 39.5]	38.2 [35.8 – 39.0]	**0.011**
Initial Glasgow coma scale	15 [13-15]	15 [14-15]	15 [12-15]	0.018
Initial blood lactate (mmol.l^−1^)	6.2 ± 3.7	5.8 ± 3.5	6.9 ± 4.0	0.059
Fluid expansion (ml)	625 [500 – 1200]	675 [500 – 1000]	500 [500 – 1000]	0.762
Fluid expansion / body weight (ml.kg^−1^)	15 ± 10	15 ± 10	14 ± 10	0.812
Norepinephrine administration	100 (25%)	83 (29%)	32 (28%)	0.357
Norepinephrine dose (mg.h^−1^)	1.0 [0.5 – 2.0]	1.0 [0.5 – 2.0]	1.0 [0.7 – 2.0]	0.668
Prehospital AB administration	114 (28%)	83 (29%)	31 (27%)	0.701
Prehospital duration (min)	65 ± 32	64 ± 32	66 ± 31	0.373
***Prehospital final values***				
Final SBP (mmHg)	103 ± 26	104 ± 26	100 ± 28	0.147
Final DBP (mmHg)	60 ± 18	60 ± 18	58 ± 20	0.360
Final MBP (mmHg)	74 ± 20	74 ± 19	72 ± 22	0.242
Final HR (beats.min^−1^)	106 ± 25	107 ± 24	109 ± 27	0.267
Final SI	1.1 ± 0.7	1.1 ± 0.7	1.1 ± 0.4	0.595
Delta SI	0.1 ± 0.7	0.01 ± 0.8	0.01 ± 0.5	0.886
Positive delta SI (%)	248 (61%)	183 (63%)	65 (56%)	0.188
Negative delta SI (%)	158 (39%)	107 (37%)	51 (44%)	0.188
Final RR (movements.min^−1^)	25 [20-33]	24 [18-30]	29 [22-35]	**0.002**
Final pulse oximetry (%)	97 [94 – 99]	97 [95 – 99]	96 [92 – 98]	**0.004**
Final body core temperature (°C)	38.2 [37.0 – 39.0]	38.3 [37.2 – 39.0]	37.8 [35.9 – 38.9]	**0.004**
Final Glasgow coma scale	15 [14-15]	15 [14-15]	15 [13-15]	**0.001**
Final blood lactate (mmol.l^−1^)	4.5 ± 3.6	3.7 ± 3.0	6.1 ± 4.3	** < 10**^**–3**^
Delta blood lactate (mmol.l^−1^)	1.2 ± 2.8	1.6 ± 2.9	0.4 ± 2.5	**0.01**
Positive delta blood lactate (%)	115 (28%)	80 (28%)	35 (30%)	0.161
Negative delta blood lactate (%)	54 (13%)	32 (11%)	22 (19%)	0.217
***Hospital parameters***				
SOFA score	7 [3-10]	6 [3-9]	9 [6-12]	** < 10**^**–3**^
SAPS2 score	58 ± 22	52 ± 19	70 ± 22	** < 10**^**–3**^
In-ICU length of stay (days)	5 [2-9]	5 [2-9]	4 [2-8]	0.099
In-hospital length of stay (days)	12 [7-20]	15 [9-24]	4 [2-13]	** < 10**^**–3**^
***Presumed septic shock origins***				
Pulmonary	170 (42%)	111 (38%)	59 (51%)	**0.021**
Digestive	102 (25%)	70 (24%)	32 (28%)	0.470
Urinary	67 (17%)	56 (19%)	11 (9%)	**0.018**
Cutaneous	28 (7%)	23 (8%)	5 (4%)	0.200
Meningeal	9 (2.5%)	7 (2%)	2 (2%)	0.671
Gynaecological	3 (0.5%)	3 (100%)	0 (0%)	0.986
Ears nose throat	2 (0.5%)	1 (50%)	1 (50%°	0.516
Endocarditis	2 (0.5%)	2 (100%)	0 (0%)	0.983
Unknown	23 (5%)	17 (6%)	6 (5%)	0.786

*SBP* Systolic blood pressure, *DBP* Diastolic blood pressure, *MBP* Mean blood pressure, *HR* Heart rate, *RR* Respiratory rate, *SI* Shock index, *ICU* Intensive care unit, *SOFA* Sequential organ failure assessment, *SAPS*2 Simplified acute physiology score 2^nd^ version, *HI*V Human immunodeficiency virus, *COPD *Chronic obstructive pulmonary disease, *AB* Antibiotic therapy, *min* Minutes, delta SI = Initial SI-Final SI, negative delta SI = Initial SI-Final SI < 0, positive delta SI = Initial SI-Final SI > 0, delta Lactate = Lactate SI-Lactate SI, negative delta Lactate = Initial Lactate -Final Lactate < 0, positive delta Lactate = Initial Lactate -Final Lactate > 0.

*Values in bold indicate a p-value* < *0.05 between living and deceased patients*

The median length of stay in a hospital was 12 [7–20] days and the median ICU length of stay was 5 [2–9] days (*Table **1*).

Pulmonary, digestive and urinary infections were the suspected cause of the SS in 42%, 25% and 17% of the cases, respectively (*Table **1*).

The 28-day overall mortality rate reached 29%.

No significant difference in the duration of the prehospital stage was observed between patients who survived and those who died on day-28 (64 ± 32 min vs 67 ± 31 min, *p* > 0.05; *Table **1*).

One-hundred and fourteen patients (28%) received prehospital antibiotic therapy, no significant difference was observed between patients who survived (*n *= 83, 29%) and those who died on day-28 (*n* = 31, 27%—*p* = 0.701) (*Table **1*). Among the 114 patients (28%) who received antibiotics prior to hospital admission, 74% were treated with 3^rd^ generation cephalosporin, 42% with cefotaxime and 31% with ceftriaxone.

All patients received crystalloids infusion for hemodynamic optimization in the prehospital setting. No significant difference in prehospital fluid expansion was observed between alive and deceased patients on day-28: 675 [500 – 1000] vs 500 [500 – 1000] ml respectively, *p* = 0.762) (*Table **1*).

One hundred patients (25%) received norepinephrine in the prehospital setting, among which 32 patients (28%) were deceased and 83 patients (29%) were alive on day-28 (*p* = 0.357). No other catecholamine was used in the prehospital setting.

No significant difference was observed for norepinephrine dose between alive and deceased patients on day-28: 1.0 [0.5 – 2.0] vs 1.0 [0.7 – 2.0] mg.h^−1^ respectively, *p* = 0.668 (*Table **1*).

Bivariate analysis reported a significant association between 28-day mortality and age, prehospital initial and final body core temperature, final prehospital respiratory rate, pulse oximetry, blood lactate, delta blood lactate, Glasgow coma scale, SOFA score, IGS-2 score and in-hospital length of stay (*Table **1*).

### Main measurement

In the overall population, the mean initial SI was 1.2 ± 0.5, the mean final SI was 1.1 ± 0.7 and the mean delta SI was 0.1 ± 0.7. Two hundred and forty-eight patients (61%) had a positive delta SI, and 158 patients (39%) had a negative delta SI. Comparisons between patients with a positive and a negative delta SI are reported in ***Table ******2****.*

**Table 2:** Comparison between patients with a positive and a negative delta SI. Results were expressed as mean and standard deviation for quantitative parameters (normal distribution), as median and interquartile range for quantitative parameters (non-gaussian distribution) and, as absolute value and percentage for qualitative parameters. P-value corresponds to the comparison between deceased and living patients

	***Positive SI (n* = *249)***	***Negative SI (n* = *157)***	*p value*
***Demographics***			
Age (years)	70 ± 15	68 ± 16	0.247
Hypertension	102 (41%)	64 (41%)	0.968
Chronic cardiac failure	33 (13%)	36 (23%)	**0.012**
Diabetes Mellitus	68 (27%)	38 (24%)	0.488
Cancer history	88 (35%)	56 (36%)	0.946
COPD	30 (12%)	25 (16%)	0.268
Chronic Renal Failure	32 (13%)	20 (13%)	0.974
***Prehospital initial values***			
Initial SBP (mmHg)	91 ± 26	115 ± 31	** < 10**^**–3**^
Initial DBP (mmHg)	53 ± 17	69 ± 21	** < 10**^**–3**^
Initial MBP (mmHg)	65 ± 19	83 ± 22	** < 10**^**–3**^
Initial HR (beats.min^−1^)	117 ± 28	106 ± 27	**10**^**–4**^
Initial RR (movements.min^−1^)	28 [22-36]	31 [24-40]	0.135
Initial pulse oximetry (%)	93 [88 – 97]	92 [84 – 96]	0.139
Initial body core temperature (°C)	38.5 [37.0 – 39.4]	38.4 [37.0 – 39.3]	0.707
Initial Glasgow coma scale	15 [13-15]	15 [14-15]	0.168
Initial blood lactate (mmol.l^−1^)	6.0 ± 3.6	6.8 ± 4.0	0.212
Fluid expansion (ml)	750 [500 – 1250]	500 [500 – 1000]	** < 10**^**–3**^
Fluid expansion / body weight (ml.kg^−1^)	16 ± 11	12 ± 7	**10**^**–4**^
Norepinephrine administration	75 (30%)	25 (16%)	**10**^**–3**^
Norepinephrine dose (mg.h^−1^)	1.0 [0.6 – 2.0]	1.0 [0.5 – 2.0]	0.676
Prehospital AB administration	80 (32%)	34 (22%)	**0.023**
Prehospital duration (min)	70 ± 31	56 ± 30	** < 10**^**–3**^
***Prehospital final values***			
Final SBP (mmHg)	110 ± 25	92 ± 25	** < 10**^**–3**^
Final DBP (mmHg)	63 ± 19	54 ± 16	** < 10**^**–3**^
Final MBP (mmHg)	78 ± 20	67 ± 18	** < 10**^**–3**^
Final HR (beats.min^−1^)	104 ± 25	110 ± 26	**0.010**
Final RR (movements.min^−1^)	24 [19-32]	28 [21-35]	**0.022**
Final pulse oximetry (%)	97 [95 – 99]	97 [93 – 99]	0.198
Final body core temperature (°C)	38.2 [36.9 – 39.0]	38.1 [37.0 – 39.0]	0.900
Final Glasgow coma scale	15 [14-15]	15 [14-15]	0.149
Final blood lactate (mmol.l^−1^)	4.4 ± 3.6	4.5 ± 3.7	0.823
Delta blood lactate (mmol.l^−1^)	1.1 ± 2.8	1.3 ± 2.7	0.659
Positive delta blood lactate	81 (32%)	34 (22%)	0.994
Negative delta blood lactate	37 (15%)	17 (11%)	0.708
***Hospital parameters***			
SOFA score	7 [4-10]	8 [2-10]	0.501
SAPS2 score	56 ± 21	61 ± 23	0.115
In-ICU length of stay (days)	5 [3-8]	4 [2-9]	0.326
In-hospital length of stay (days)	12 [7-20]	11 [6-20]	0.258

*SBP* Systolic blood pressure, *DBP* Diastolic blood pressure, *MBP* Mean blood pressure, *HR* Heart rate, *RR* Respiratory rate, *SI* Shock index, *ICU* Intensive care unit, *SOFA* Sequential organ failure assessment, SAPS2 = simplified acute physiology score 2^nd^ version, COPD = chronic obstructive pulmonary disease, AB = antibiotic therapy, min = minutes, negative delta SI = Initial SI—Final SI < 0, positive delta SI = Initial SI—Final SI > 0, delta Lactate = Lactate SI—Lactate SI, negative delta Lactate = Initial Lactate—Final Lactate < 0, positive delta Lactate = Initial Lactate—Final Lactate > 0.

*Values in bold indicate a p-value* < *0.05*

Conversely, the mean initial blood lactate was 0.02 ± 0.04 mmol.l^−1^.min^−1^, the mean final blood lactate was 0.02 ± 0.05 mmol.l^−1^.min^−1^ and the mean delta blood lactate was 0.002 ± 0.1 mmol.l^−1^.min^−1^ (0.002 ± 0.1 for alive patients and 0.001 ± 0.1 mmol.l-1.min-1 for alive and deceased patients respectively, *p* = 0.215). One-hundred and fifteen patients (28%) had a positive delta blood lactate, and 54 patients (13%) had a negative delta blood lactate.

**Fig. 1:**
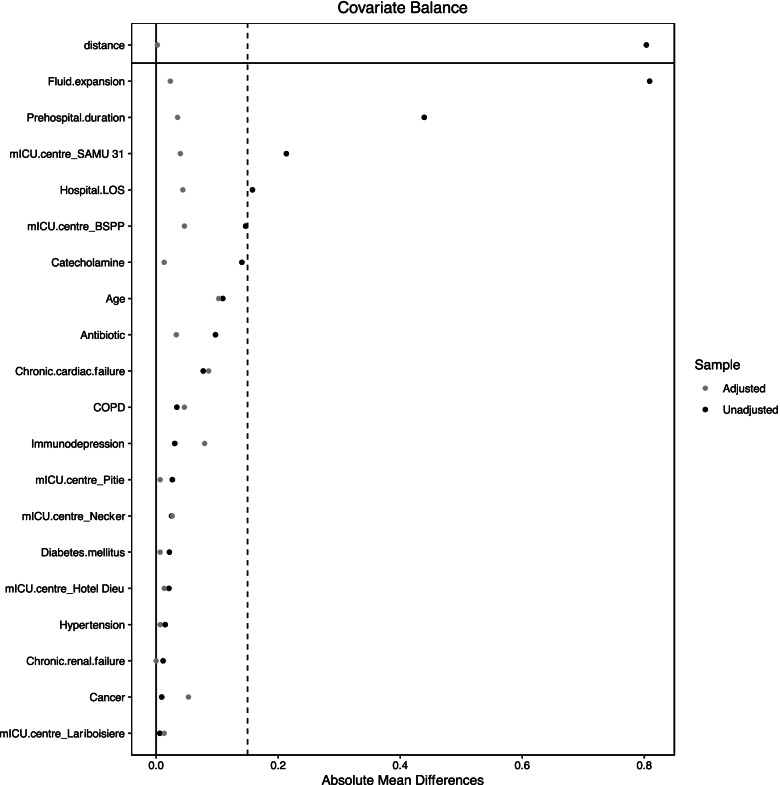
Absolute mean differences between patients with prehospital hemodynamic optimisation achievement and those without prehospital hemodynamic optimisation achievement after matching

After propensity score matching for positive SI, 337 patients: i.e., 240 negative delta SI and 97 positive delta SI were compared. Comparisons are reported in *Table **3* and the absolute mean differences between subgroups after propensity score matching are depicted in *Fig. **1*.

**Table 3:** Comparison of predictive variable for 28-day mortality included in the propensity score before and after matching. Values are expressed as mean ± SD or number (%). d corresponds to the standard mean deviation

**Positive SI**	Before Matching *n* = 406			After Matching *n* = 337		
PS covariate	Cases	Controls	*p* value (d*)	Cases	Controls	*p* value (d*)
	*n* = 157	*n* = 249		*n* = 97	*n* = 240	***0.01***
Age	68 ± 16	70 ± 15	0.24	72 ± 14	67 ± 16	0.92
Hypertension	65 (41%)	101 (41%)	0.93	41 (42%)	100 (42%)	0.16
COPD	25 (16%)	31 (12%)	0.29	16 (16%)	26 (11%)	0.16
Cancer	56 (35%)	88 (35%)	0.99	74 (76%)	40 (17%)	0.07
Diabetes mellitus	39 (25%)	68 (27%)	0.60	22 (23%)	72 (30%)	0.18
Chronic cardiac failure	36 (23%)	33 (13%)	0.01	20 (21%)	34 (14%)	0.15
Chronic renal failure	20 (13%)	33 (13%)	0.94	17 (18%)	26 (11%)	0.09
Immunodepression	43 (27%)	77 (31%)	0.46	29 (30%)	64 (27%)	0.55
Fluid expansion	500 [500–1000]	750 [500–1250]	< 10^–3^	750 [500–1200]	750 [500–1025]	0.71
Catecholamine	25 (16%)	77 (31%)	0.001	29 (30%)	62 (26%)	0.45
Antibiotic therapy	35 (22%)	80 32%)	0.03	25 (26%)	77 (32%)	0.26
Prehospital duration	56 ± 30	70 ± 31	< 10^–3^	60 ± 30	66 ± 32	0.50
Hospital LOS	11 [6-21]	12 [7-20]	0.27	6 [2-12]	15 [9-23]	** < *10***^***–3***^

*PS* Propensity score, *LOS* Length of stay, *COPD* Chronic obstructive pulmonary disease

Using Cox regression analysis on matched population, we observed a significant association between 28-day mortality and negative delta SI with an adjusted hazard ratio (HRa) of 1.88 [1.07–3.31] (*p* = 0.03) as for positive delta SI: HRa = 0.53 [0.30–0.94] (*p* < 10^–3^).

**Fig. 2:**
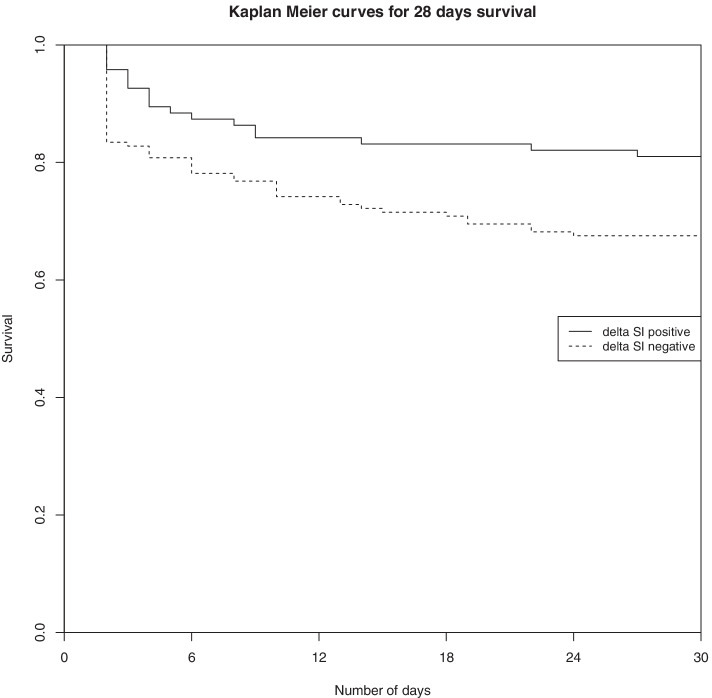
Kaplan Meier curves of 28-day survival between patients with negative SI and those with positive SI

Figure 2 depicts Kaplan Meier curves after confounder adjustment for 28-day survival between positive delta SI and negative delta SI patients (*Fig. **2*).

**Fig. 3:**
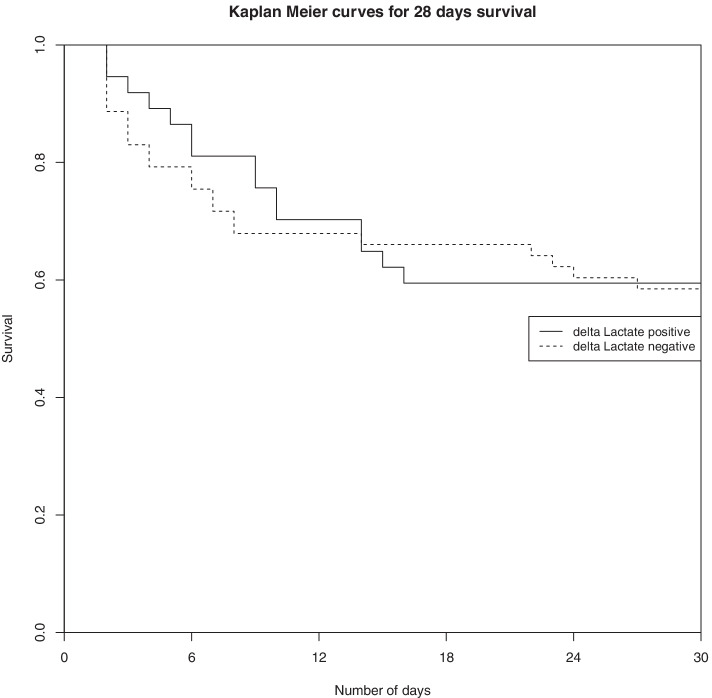
Kaplan Meier curves of 28-day survival between patients with negative delta lactate and those with positive delta lactate

Figure 3 represents Kaplan Meier curves after confounder adjustment for 28-day survival between positive delta lactate and negative delta lactate patients (*Fig. **3*).

## Discussion

In this study, we observed a significant association between 28-day mortality and prehospital delta shock index. Negative prehospital delta SI is associated with a 1.9-fold 28-day mortality increase whereas positive prehospital delta SI is associated with a 1.9-fold 28-day mortality decrease among septic shock patients cared for by a mICU in the prehospital setting.

In order to reduce mortality related to sepsis, the World Health Assembly, the World Health Organization and the “SEPSIS-3” conference recommand early sepsis recognition and severity assessment, as key elements prior to early treatment initiation [7, 8].

In-hospital studies reported an association between septic shock patient poor outcome and clinical signs, biomarkers and severity scores, i.e., SOFA and IGS-2 [29, 30, 34–37]. Nevertheless, in the prehospital setting, only clinical signs and few biomarkers are available [12]. Elsewhere, the qSOFA score, which do not require biological criteria as opposed to SOFA and IGS-2, has been suggested for assessing sepsis severity, but its validity remains under debate [38–44]. To date, for sepsis severity assessment, blood lactate is the best biomarker [45, 46], available in the prehospital setting [28], associated with survival [15, 28, 47, 48]. In addition, lactate clearance represents an indirect tool for treatment effect assessment [7, 21], usable to guide sepsis resuscitation [7, 19, 20], although subjected to controversies [49, 50]. However, blood lactate point of care testing is not worldwide available in the prehospital setting.

To bypass biomarkers’ limits, clinical signs have been proposed. On the one hand, capillary refill time and skin mottling score are associated with 28-day mortality [12]. SI, a simple clinical objective [51] tool, has an higher ability than hemodynamic physical signs (heart rate and blood pressure) for septic shock severity assessment [10] and is usable for early triage [13, 52]. To the best of our knowledges, this is the first study reporting a dynamic analysis of the SI in the prehospital stage of septic shock patients cared for by a mICU.

Nevertheless, the current study suffers from several limitations restricting the conclusions generalization. We cannot rule out a bias from misclassification of covariates, because data were collected from prehospital and in-hospital medical reports. Data accuracy may be compromised because data abstraction was collected by a single investigator [53]. Patients were only adults, consequently, the conclusions are not directly transposable to pediatric populations. Beta-blockers are widely prescribed, limiting SI increase despite an underlying illness. Despite no significant difference between alive and deceased patients, the fluid volume expansion is lower than recommended [7]. The study is retrospective; thus, no therapeutic goal (antibiotic therapy and/or hemodynamic optimization) was required for the mICU teams. In addition, the study focused on patients with septic shock, not on sepsis or other shock-etiologies. The external validity is affected by the specificity of the French prehospital EMS, based on SAMU and mICU intervention in the prehospital setting, contrary to others prehospital EMS organization based on paramedics.

Beyond these limitations, in a similar manner to lactate clearance, the ability of delta SI (i) to be an indirect tool for treatment effect assessment, and (ii) enabling sepsis resuscitation guiding, requires larger prospective trials.

## Conclusion

Delta shock index in the prehospital stage of septic shock patients cared for by a mICU is significantly associated with 28-day mortality. A negative prehospital delta SI is associated with a 1.9-fold 28-day mortality increase whereas positive delta SI is associated with a 1.9-fold 28-day mortality decrease. Further studies are needed to evaluate the ability of prehospital delta SI to assess hemodynamic optimization treatment effect assessment and its usefulness for sepsis resuscitation guiding during the prehospital setting.

## Authors' information

not applicable

## Data Availability

The dataset analyzed during the current study are not publicly available because their containing information that could compromise the privacy of *research* participants but are available from the corresponding author on reasonable request.

## Abbreviations

SS: Septic shock
SI: Shock index
mICU: Mobile intensive care unit
HRa: Adjusted hazard ratio
ED: Emergency department
ICU: Intensive care unit
SAMU: Urgent Medical Aid Service
SMUR: Mobile Emergency and Resuscitation Service
SBP: Systolic blood pressure
DBP: Diastolic blood pressure
MAP: Mean arterial pressure
HR: Heart rate
SpO2: Pulse oximetry
RR: Respiratory rate
GCS: Glasgow coma scale
LOS: Length of stay
SOFA: Sequential Organ Failure Assessment
SAPS 2: Simplified Acute Physiology Score
SMD: Standardized mean deviation
